# Supplementary material for: In vivo expansion of functionally integrated GABAergic interneurons by targeted increase in neural progenitors
Source: EMBO J. 2018 May 4;37(13):e98163. doi: 10.15252/embj.201798163 (PMC6028031; doi:10.15252/embj.201798163)
Supplement: Supplementary file 1 — Expanded View Figures PDF [file EMBJ-37-e98163-s001.pdf]

Expanded View Figures

Figure EV1. Unlike moderate Pros knock-down, *pros* and *mira* mutations are tumorigenic though only when induced in early larval NSCs (related to Fig 2).
MARCM clones were induced by heat-shock (hs) at either early L2 (EL2; "induced early") or ML3 ("induced late") stages and animals were allowed to develop up to either WL3 or adult stages, at which times they were dissected and incubated with EdU or fixed for immunohistochemistry. All clone pictures are single optical sections in which clones are outlined with dotted lines in split channels.

A Pictures of WL3 MARCM clones induced early. Unlike WT clones, which contain a single NSC (identifiable by larger size and expression of Dpn) and mostly neurons (labeled by Elav), *pros*-null clones predominantly contained Dpn^{+} cells and were usually devoid of neurons.

B Pictures of adult MARCM clones induced early. Unlike WT clones, in which EdU, Dpn, and Chinmo are never seen at adult stages, *pros*-null clones contained ectopic adult NSCs, labeled by Dpn, which were able to incorporate EdU, and including ones expressing the early temporal marker Chinmo.

C Tumorigenesis assay: WL3 ventral nerve cord (VNC) fragments containing GFP-labeled MARCM clones induced early were injected into abdomen of adult WT hosts, which resulted in invasion of the entire abdomen by *pros*-null GFP^{+} cells in more than 50% cases ("positive"), and even detection of GFP^{+} cells at distant locations (arrow), which was never seen when WT GFP^{+} cells were injected.

D Experimental design to assess tumorigenicity of *pros* and *mira* mutations induced in neural lineages at early versus late larval stages. Blue lines: developmental period between clone induction and CNS fixation. VNC clones (thoracic region outlined in dashed lines) were analyzed to ensure scoring of type I NSC clones.

E WL3 clones of two independent alleles of either *pros* or *mira* were scored as: i. WT-looking (i.e., containing a single Dpn^{+} cell and a few Dpn^{-} progeny); ii. stalled NSC (i.e., containing a single Dpn^{+} cell and no progeny, suggesting cell-cycle delay or stalling); iii. ectopic NSCs (i.e., containing multiple Dpn^{+} cells). Dpn^{-} clones were excluded from the analysis. Histograms of clone category proportions per CNS (n = 7–12 CNSs; details in source data) show that late clone induction reduces the number of clones with ectopic NSCs in *pros* hypomorphic conditions such as in $pros^{1L32}$ (see (G) for molecular information) and for two *mira*-null alleles [expected to behave analogously to a *pros* hypomorph since Mira depletion from NSCs presumably results in diminished GMC inheritance of Pros (which in some cases is followed by its reversion to NSC, hence tumorigenicity of *mira* mutants)]. Statistical significant difference between number of clones induced early or late (for the same genotype) that contained ectopic NSCs is indicated: $^{**}P < 0.05$, $^{***}P < 0.001$ (Mann–Whitney test). Error bars SEM.

F Pictures of adult MARCM clones co-stained for EdU. Only clones induced early led to ectopic proliferation in the adult brain, indicating that ectopic NSCs visible at WL3 in clones induced late terminated proliferation prior to adult stage and are therefore non-tumorigenic (n = 20 for each condition).

G Molecular characterization of the new hypomorphic $pros^{1L32}$ allele (that survives to L1) uncovered in a forward genetic screen (R.S.N., W. Greg Somers and W. Chia, unpublished). Schematic representation of *pros* mRNA isoforms: Each box represents an exon; untranslated regions are in gray; and coding sequence is in white; in orange is the site of a C-to-T mutation—in a coding region common to all isoforms and that converts a Gln (Q) residue (position 859 in isoforms H, I, and M) to a premature STOP codon (*), resulting in severely truncated proteins.

Data information: See main text for other abbreviations.
Source data are available online for this figure.

© 2018 The Authors

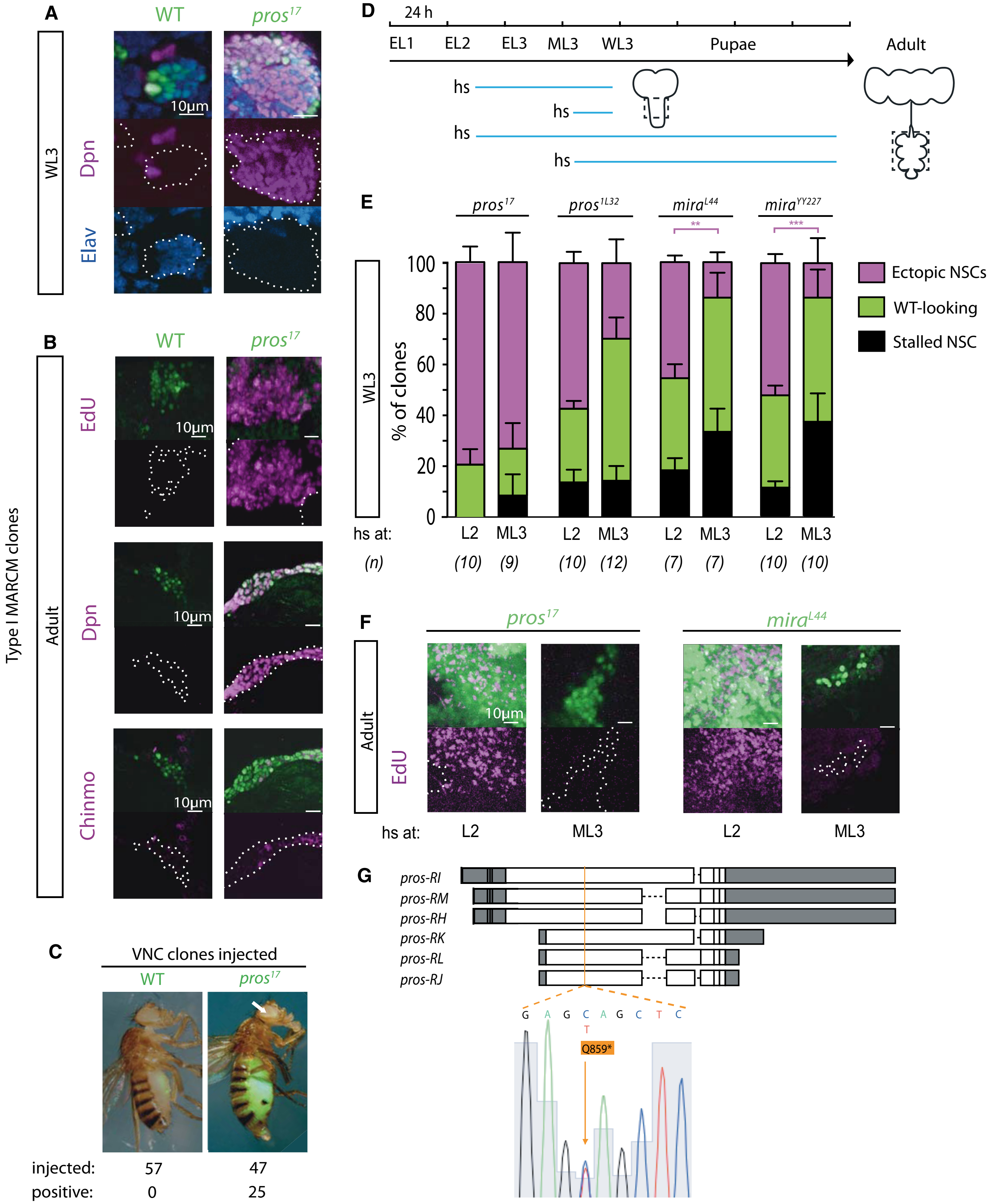

Figure EV1.

© 2018 The Authors

Figure EV2. Two RNAi lines that target *pros* with different strengths lead to NSC expansion from early larval stages (related to Fig 2).

A Schematic representation of *pros* mRNA isoforms: Each box represents an exon; untranslated regions are in gray; and coding regions are in white; in red are the target regions for $prosRNAi^{LH}$ and $prosRNAi^{KK}$, both of which target all isoforms.

B Pictures of WL3 stage DAL lineages (outlined with dashed lines in split magenta channel) in WT or in animals in which Pros has been knocked down with either RNAi at 22°C. Lineages with $prosRNAi^{LH}$ knock-down contain a few $Pros^{+}$ cells in contrast to lineages with $prosRNAi^{KK}$ knock-down, in which no $Pros^{+}$ cells are detectable and which are larger (single optical sections of stainings performed in parallel and imaged with same settings). In line with the functional data presented in main Fig 2 (panel E), this demonstrates that the two RNA is specifically target *pros* but that $prosRNAi^{LH}$ is weaker than $prosRNAi^{KK}$.

C Quantification of Dpn^{+} and Dpn^{-} cells in DAL lineages in the conditions indicated; histograms represent the mean (n = 5–6 CNSs; details in source data) and error bars SEM. Compare with Fig 2B.

D Pictures of permanently labeled DAL lineages (outlined in dashed lines in split magenta channel; maximum intensity projections) at adult stage, expressing $prosRNAi^{LH}$ under the strong induction of *actin5C-GAL4*. Unlike in animals reared at 22°C, in which EdU was never seen, stronger *pros* depletion at 25°C results in clones with ectopically replicating cells (boxed area shown at higher magnification in inset). Note that the *actin5C* promoter responsible for permanent labeling leads to significantly higher levels of expression than the *engrailed* regulatory sequence. Compare with Fig 2E.

E Time course of Dpn^{+} and Dpn^{-} cells in DAL lineages in the conditions indicated; histograms represent the mean (n = 4–13 CNSs; details in source data) and error bars SEM. Unlike in controls (driver animals reared at 25°C), non-tumorigenic expansion using $prosRNAi^{LH}$ results in ectopic NSCs already from EL3 stage.

Data information: See main text for abbreviations.
Source data are available online for this figure.

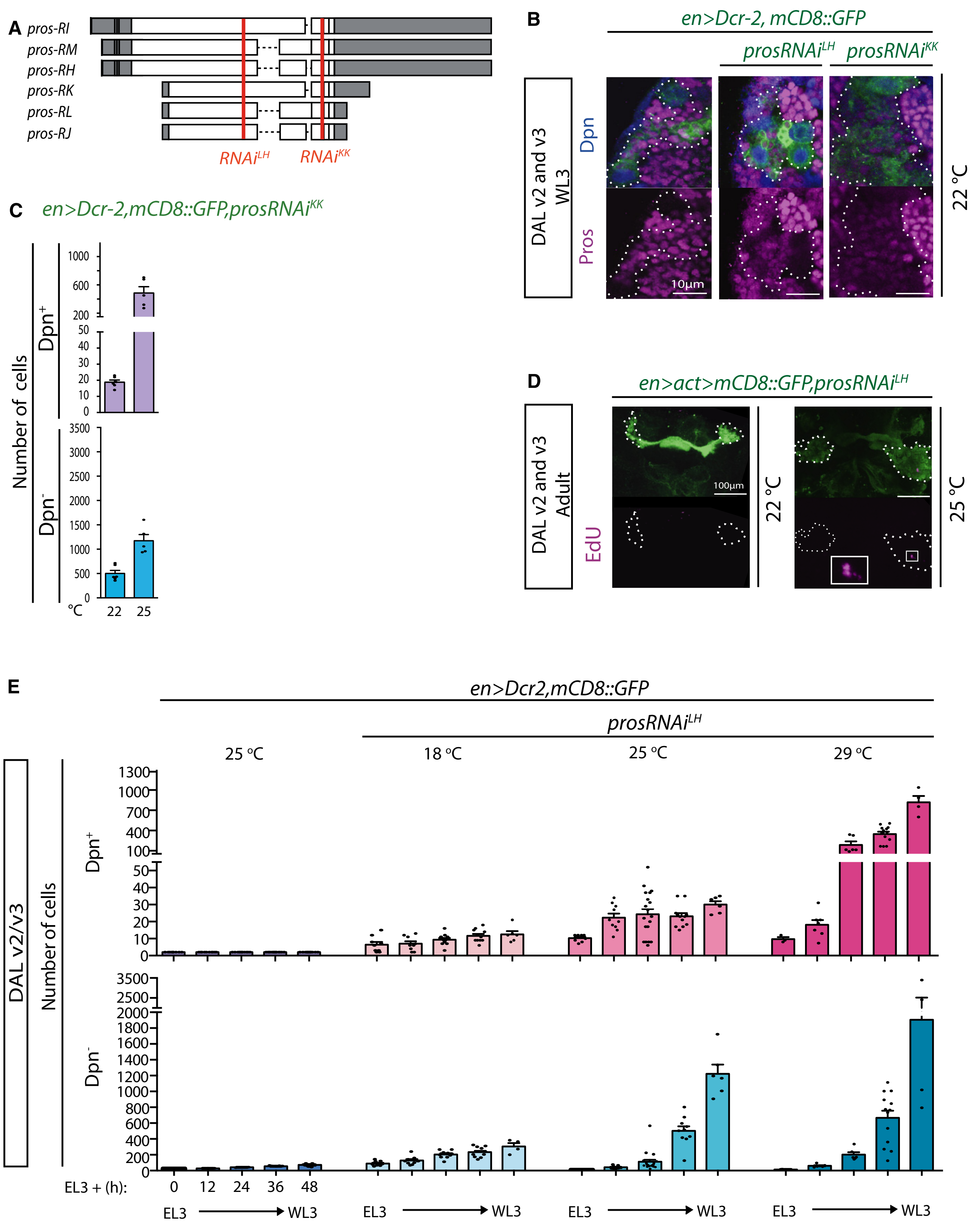

Figure EV2.

 © 2018 The Authors

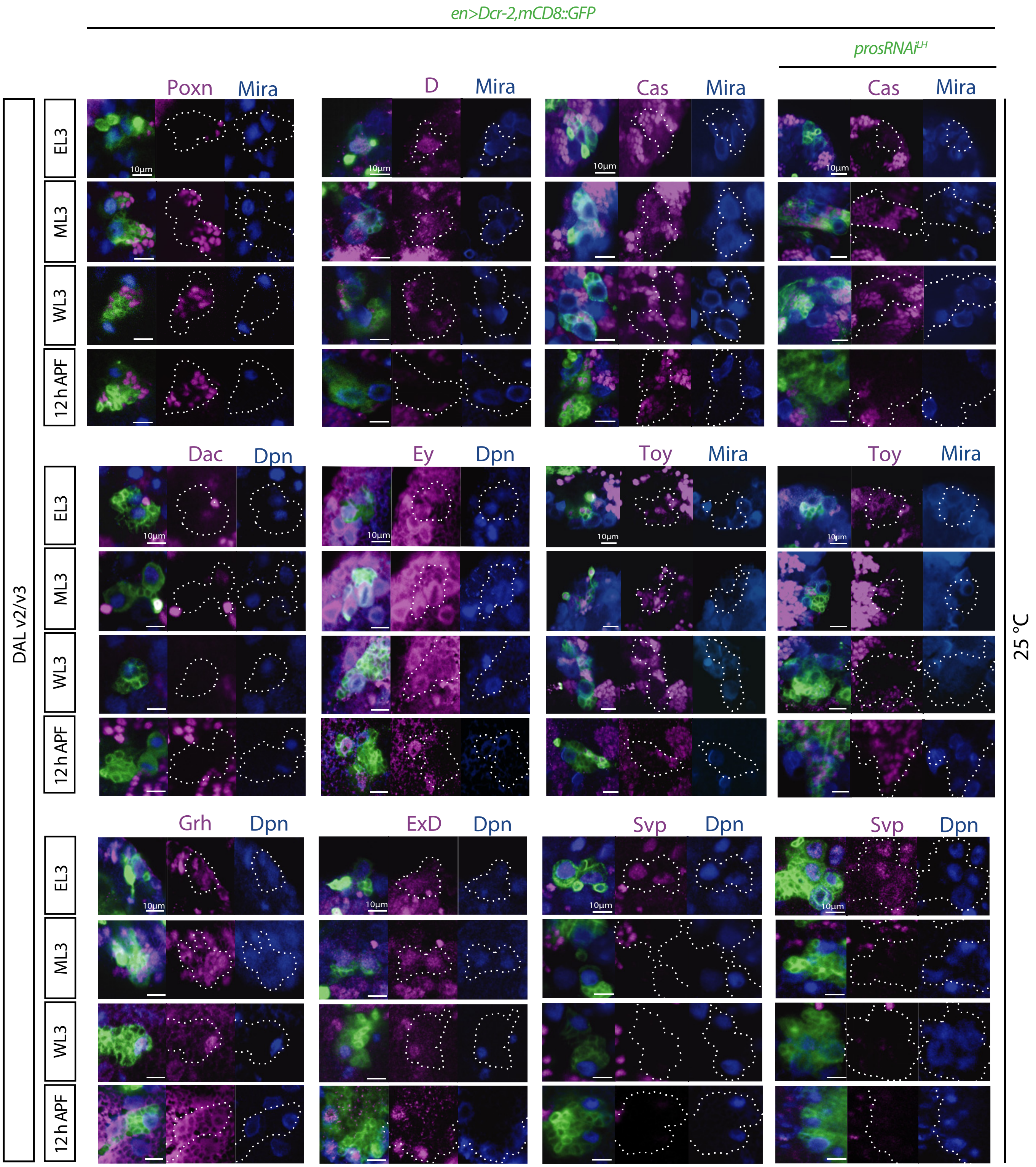

Figure EV3. Time course of marker expression in WT and expanded DAL lineages (related to Fig 3).

Pictures of expression time course of the temporal markers Poxn, D, Dac, Ey, Grh, and ExD in WT DAL lineages, and Cas, Toy, and Svp in WT and expanded DAL lineages (single optical sections; lineages outlined in dashed lines in split magenta and blue channels). Co-staining for Mira or Dpn allows identification of the NSCs. Poxn, D, Dac, Toy and Svp were temporally restricted within this time-window; Cas was never detected in NSCs within this time-window; Ey, Grh, and ExD were expressed throughout these timepoints. See main text for abbreviations.

© 2018 The Authors

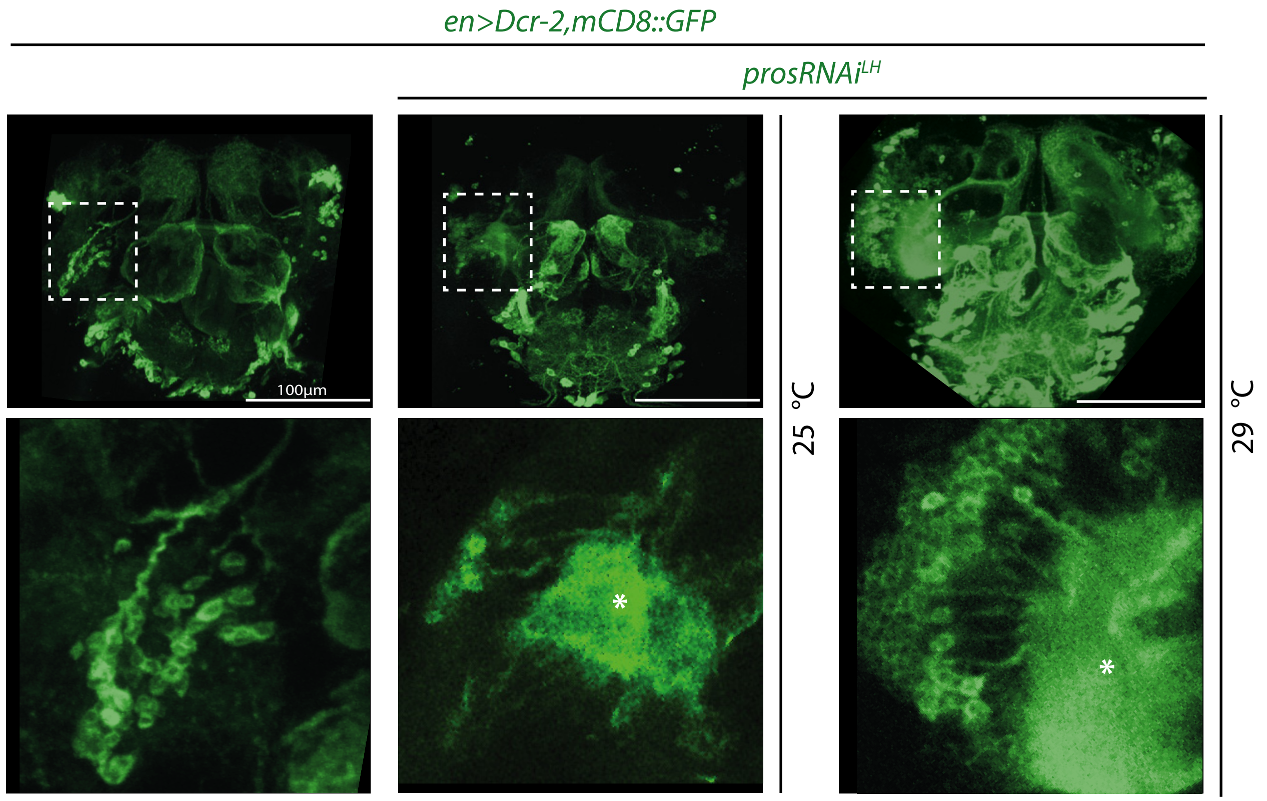

Figure EV4. Expansion of DAL neurons can result in ectopic neuropil formation (related to Fig 4).

Pictures of en>GFP-labeled adult central brains (maximum intensity projections; top row) that are either WT (left) or expressing *prosRNAi*^{LH} at 25°C or 29°C. DAL lineages are outlined with dashed lines and magnified in bottom row (single optical sections). Asterisks indicate ectopic neuropil. See main text for abbreviations.

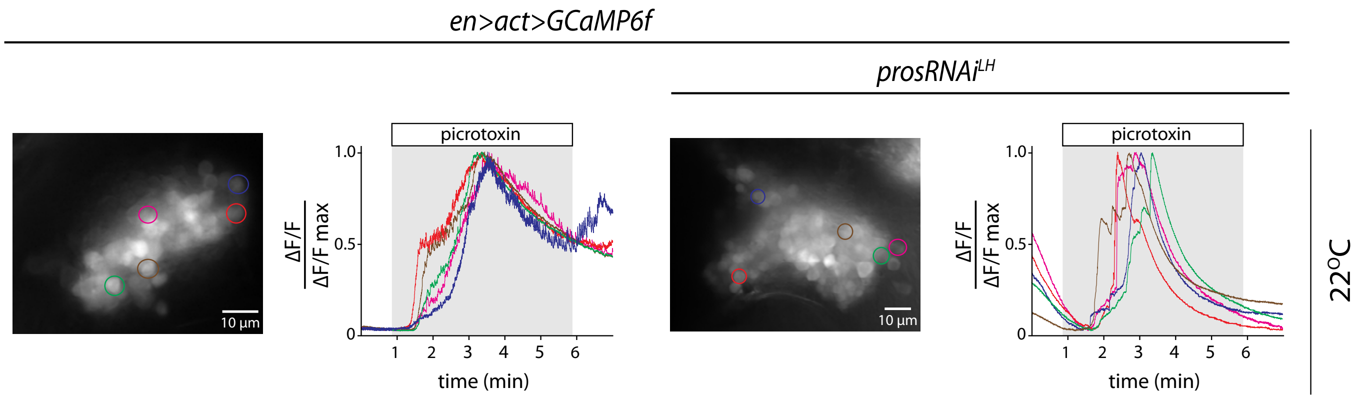

Figure EV5. Additional calcium responses of DAL neurons and supernumerary DAL neurons to picrotoxin (related to Fig 4).

Maximum intensity projections of GCaMP6f expression in adult DAL lineages of WT and expanded DAL lineages with *pros* knock-down over 7 min of recording, including response to picrotoxin (added at 1 min and washed out at 6 min: gray background), with color outline of five exemplary cells per genotype whose individual normalized calcium traces are shown. There is biological variability in both control and *pros* knock-down brains, which we tried to convey in the figures by depicting a range of responses. Again, we could not detect any individual neuron that did not respond to the picrotoxin treatment suggesting that supernumerary DAL neurons are functional.

 © 2018 The Authors

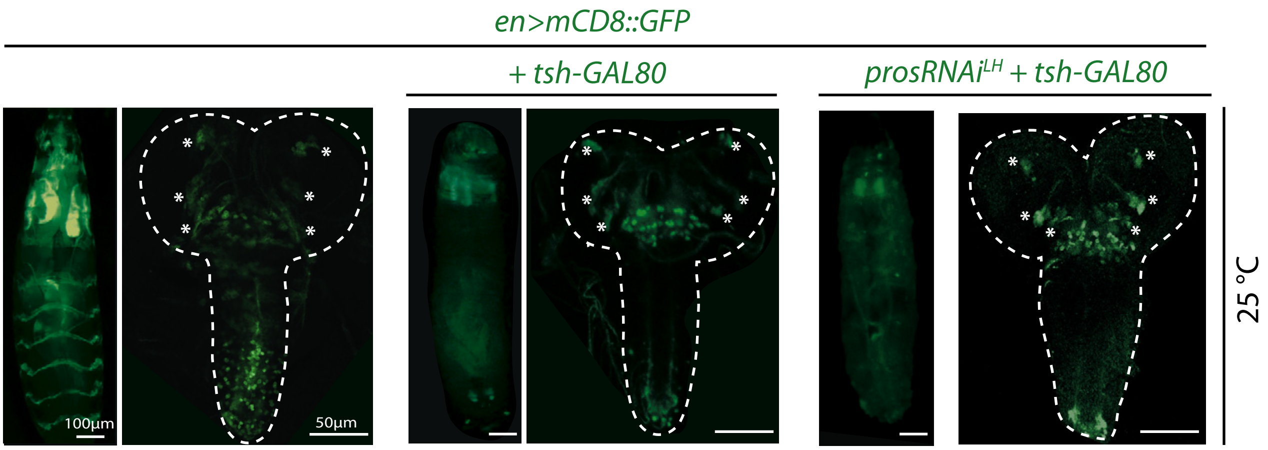

Figure EV6. Adult DAL lineages expand in the absence or presence of *tsh-GAL80* (related to Fig 5).

Pictures of whole WL3 larvae (left for each genotype) and of whole WL3 CNSs (right for each genotype) of animals raised at 25°C. *tsh-GAL80* greatly dampens en>GFP expression in trunk segments (thorax and abdomen excluding the VNC tip) but not cephalic ones (including DAL lineages, marked with asterisks). See main text for abbreviations.
