# Supplementary material for: In vivo expansion of functionally integrated GABAergic interneurons by targeted increase in neural progenitors
Source: EMBO J. 2018 May 4;37(13):e98163. doi: 10.15252/embj.201798163 (PMC6028031; doi:10.15252/embj.201798163)
Supplement: Supplementary file 2 — Movies EV1–EV6 [file EMBJ-37-e98163-s002.zip › Shaw_et_al_EV_Movie_1_(ctr)_legend.docx]

**EV Movie 1. Related to Figure 2. Larval DAL NSCs divide asymmetrically in WT.**

DAL NSCs (large cells) of *en>act>mCD8::GFP* at WL3 stage. One NSC can be seen to divide asymmetrically (large arrowhead) giving rise to a smaller daughter (small arrowhead). Frames were acquired every 5 min and movie is played at 2 frames/s (which is 150 times the speed of image acquisition).
