# Supplementary material for: In vivo expansion of functionally integrated GABAergic interneurons by targeted increase in neural progenitors
Source: EMBO J. 2018 May 4;37(13):e98163. doi: 10.15252/embj.201798163 (PMC6028031; doi:10.15252/embj.201798163)
Supplement: Supplementary file 2 — Movies EV1–EV6 [file EMBJ-37-e98163-s002.zip › Shaw_et_al_EV_Movie_2_(exp)_legend.docx]

**EV Movie 2. Related to Figure 2. Larval supernumerary DAL NSCs can also divide asymmetrically in *en>act>mCD8::GFP,prosRNAi^LH^*.**

DAL NSCs (large cells) of *en>act>mCD8::GFP,prosRNAi^LH^* at WL3 stage. Two NSCs can be seen to divide asymmetrically (large arrowheads) giving rise to smaller daughters (small arrowheads). Frames were acquired every 5 min and movie is played at 80 frames/s (which is 150 times the speed of image acquisition).
