# Supplementary material for: In vivo expansion of functionally integrated GABAergic interneurons by targeted increase in neural progenitors
Source: EMBO J. 2018 May 4;37(13):e98163. doi: 10.15252/embj.201798163 (PMC6028031; doi:10.15252/embj.201798163)
Supplement: Supplementary file 2 — Movies EV1–EV6 [file EMBJ-37-e98163-s002.zip › Shaw_et_al_EV_Movie_3_(ctr)_legend.docx]

**EV Movie 3. Related to Figure 4. Adult R neurons and ellipsoid body in control brains.**

3D reconstruction of Z-stack through *en>act>mCD8::GFP* adult brain showing ellipsoid body ring neurons and their projections via the lateral triangles/bulb into the ring neuropil that in *Drosophila* forms a toroid-like structure.
