# Supplementary material for: In vivo expansion of functionally integrated GABAergic interneurons by targeted increase in neural progenitors
Source: EMBO J. 2018 May 4;37(13):e98163. doi: 10.15252/embj.201798163 (PMC6028031; doi:10.15252/embj.201798163)
Supplement: Supplementary file 2 — Movies EV1–EV6 [file EMBJ-37-e98163-s002.zip › Shaw_et_al_EV_Movie_4_(exp)_legend.docx]

**EV Movie 4. Related to Figure 4. Adult supernumerary R neurons and ellipsoid body in non-tumorous brains.**

3D reconstruction of Z-stack through *en>act>mCD8::GFP,prosRNAi^LH^* adult brain showing ellipsoid body ring neurons, together with supernumerary neurons, and their projections into the ring neuropil. Note the enlarged diameter of the ring neuropil indicative of added projections from supernumerary R neurons.
