# Supplementary material for: In vivo expansion of functionally integrated GABAergic interneurons by targeted increase in neural progenitors
Source: EMBO J. 2018 May 4;37(13):e98163. doi: 10.15252/embj.201798163 (PMC6028031; doi:10.15252/embj.201798163)
Supplement: Supplementary file 2 — Movies EV1–EV6 [file EMBJ-37-e98163-s002.zip › Shaw_et_al_EV_Movie_5_(ctr)_legend.docx]

**EV Movie 5. Related to Figure 4. Calcium response of DAL neurons to picrotoxin.**

Time course of the change in GCaMP6f fluorescence in the DAL population before, during, and after application of picrotoxin (500 µM for 5 min, indicated by white circle in the top left corner). During picrotoxin application, discrete regions of the preparation, corresponding to individual cell bodies, increase in fluorescence indicating activation. The brain shown is the same as in Figure 4E and is played at 80 fps (which is 10 times the speed of image acquisition).
